# Exploring female medical students’ knowledge, attitudes, practices, and perceptions related to breast cancer screening: a scoping review

**DOI:** 10.25122/jml-2023-0412

**Published:** 2023-12

**Authors:** Mirela Tomic, Maria-Lorena Vescan, Marius-Ionuţ Ungureanu

**Affiliations:** 1 Department of Public Health, Faculty of Political, Administrative and Communication Sciences, Babeş-Bolyai University, Cluj-Napoca, Romania; 2 Faculty of Medicine, Iuliu Hatieganu University of Medicine and Pharmacy, Cluj-Napoca, Romania; 3 Center for Health Workforce Research and Policy, Faculty of Political, Administrative and Communication Sciences, Babeş-Bolyai University, Cluj-Napoca, Romania

**Keywords:** medical students, breast cancer, breast self-exam, knowledge, practice, BC – breast cancer, BSE – breast self-exam, BCS – breast cancer screening, CBE – clinical breast exam, DCIS- ductal carcinoma in situ, HBMS – Health Belief Model Scale, IMPC- invasive micropapillary carcinoma, MMG – mammography, MRI- magnetic resonance imaging, NST- non-specific type carcinoma, PRISMA-ScR – Preferred Reporting Items for Systematic Reviews and Meta-Analyses extension for scoping Reviews

## Abstract

Early detection measures for breast cancer, such as breast self-exams, clinical breast exams, and mammography, have considerable benefits in effectively reducing breast cancer-related mortality. As the incidence of breast cancer is steadily increasing, it is crucial to raise awareness on early detection. This scoping review assessed the current knowledge, attitudes, practices, and perceptions of breast cancer screening among female medical students. We used the six phases of Arksey and O'Malley's framework from the Joanna Briggs Institute Manual and the Preferred Reporting Items for Systematic Reviews and Meta-Analyses extension for Scoping Reviews (PRISMA-ScR) template. Our analysis included 43 articles from Google Scholar and PubMed search engines, focusing on female medical students. Our results showed that most female medical students had a satisfactory level of knowledge about the most common signs, symptoms, and early detection methods of breast cancer. Generally, their attitude and perceptions were positive regarding breast cancer-related preventive measures. However, the level of practice was reduced. Further efforts are necessary to promote and improve the practice of breast self-examination, clinical breast exams, and mammography among female medical students. Potential interventions could include modifications to the medical curriculum and social media campaigns to enhance engagement and adoption of these practices.

## INTRODUCTION

Globally, breast cancer (BC) is the most frequently identified disease, with more than 2.3 million new diagnoses and 685,000 deaths reported in 2020. Compared to developed countries, BC mortality continues to be disproportionately more prevalent in developing nations. Because of increasing populations and aging, the burden of BC is expected to rise to about three million new diagnoses and one million deaths annually by 2040 [1]. The rise in breast cancer mortality affects most age groups and regions worldwide. However, women under 50 are at the greatest risk [2]. In some Asian and African countries, the average age for breast cancer diagnosis is more than ten years earlier than that in European and American populations. Thus, raising health awareness and developing prevention measures is fundamental to reducing the incidence of BC, which is steadily rising, particularly in the most affected regions [3].

In the context of BC becoming a real public health concern, the importance of early detection measures such as breast self-exams (BSE), clinical breast exams (CBE), and mammography (MMG) is increasing. Regular BSE is an essential first step for early diagnosis, particularly for nations with poor access to healthcare. Furthermore, regular BSE is favorably linked with early BC detection in low and middle-income countries, which leads to improved treatment results. As a result, BSE practice and prompt medical evaluation when any abnormalities are detected could be an effective strategy for the early detection of breast cancer, which could boost survival rates [4]. Moreover, BSE holds particular significance, considering that young women cannot receive an MMG, often regarded as the gold standard in detecting BC. Nevertheless, mammography appearance is typically nonspecific for invasive micropapillary carcinoma (IMPC), with magnetic resonance imaging (MRI) evaluation being the best way to diagnose this BC [5]. Early detection for these patients is extremely important, as they typically tend to have considerable lesions diagnosed at an advanced stage with a worse prognosis due to the micropapillary aspect and the extension of lymph nodes compared to individuals with other kinds of breast cancer, particularly non-specific type carcinoma (NST) [6]. In this context, BSE is viewed as an unquestionable procedure when socioeconomic circumstances are also regarded, particularly in developing nations [7].

The progression of BC in younger women tends to be more aggressive than its development in older individuals. A subset of breast tumors with common patterns of gene expression is characterized by young age at diagnosis, which is correlated with a less favorable outcome. Likewise, a major contributing factor to the high death rate among young women is the lack of BC awareness [8]. Nowadays, various treatment options are available to improve the health status of patients. A wide range of scientific evidence is available on this subject. However, exercising caution is of great importance when interpreting the findings of studies that examined the clinical relevance of HER2 status in patients with ductal carcinoma in situ (DCIS). For patients with invasive breast cancer, a HER2 assessment holds an important status. However, this poses certain threats for patients exhibiting intermediate HER2 expression and/or intra-tumoral heterogeneity. Similarly, regarding Dductal carcinoma patients, the potential advantages of HER2 targeted therapy, including the clinical fundamental shifts, remain unclear [9].

Therefore, the main aim of our review was to map evidence in the existing scientific literature about knowledge, attitudes, practices, and perceptions of female medical students concerning BC screenings and to suggest future actions to improve the status quo. Since medical students have sufficient contact with health-related content, they might serve as informational resources for non-health students and other people in society, which makes this target group of particular interest [10]. Furthermore, students' ability to inform people about BC and BSE relies on their concurrent knowledge, practices, and health beliefs [7]. Achieving the mission of increasing cancer screening uptake is also shared with nurses and midwifery students [11]. Additionally, in developing nations where access to high-quality healthcare is still limited, nurses and midwives are typically the main sources of healthcare in remote rural communities where advanced cases of BC and mortality rates are higher compared to urban settings [12]. In these contexts, it is often their responsibility to instruct other women on performing BSE correctly [13].

## MATERIAL AND METHODS

For this scoping review, we employed the six-phase framework outlined in the Joanna Briggs Institute Manual, as developed by Arksey and O'Malley, which included the following steps: (1) defining the research issue, (2) locating pertinent studies, (3) choosing surveys, (4) mapping data, (5) and (6) gathering, synthesizing, and recording the information [14]. In addressing the first phase of answering the research question, our primary goal was to investigate the level of knowledge among medical students regarding BC screenings. Secondary to this, we also focused on their attitudes, practices, and perceptions related to breast cancer screenings.

**Figure 1:**
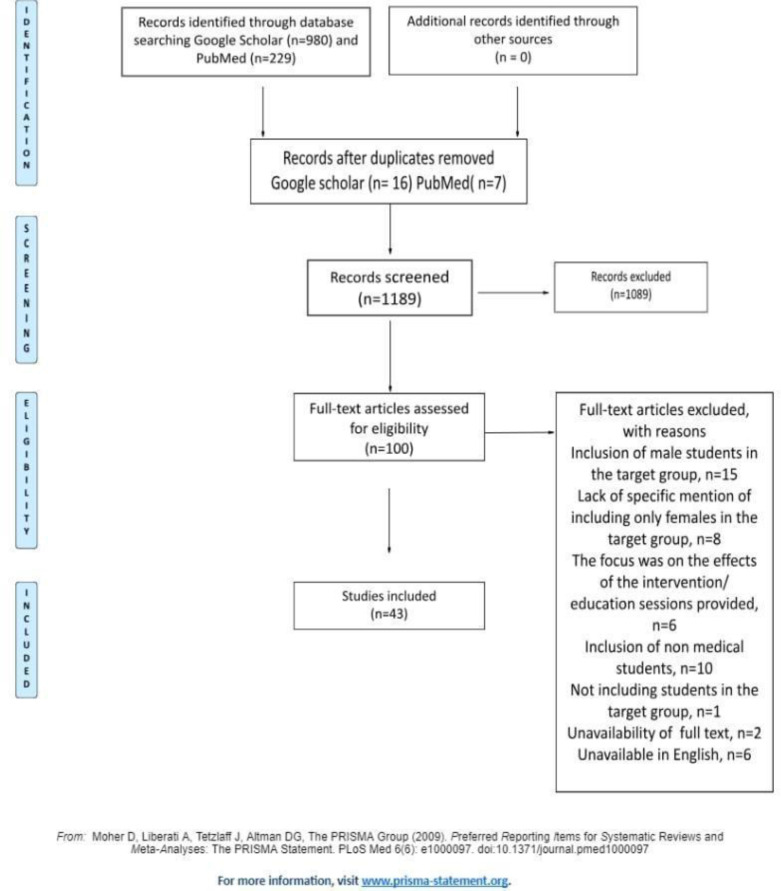
PRISMA Flow Diagram

Duplicate records were removed before storage in the reference management tool, Zotero. The first phase consisted of screening the record titles and abstracts relevant to the aim of our review. Articles that were found to be outside the reach of the review reach were not taken into consideration, specifically grey literature. The full-text review was limited to articles considered relevant by at least one reviewer based on our predefined inclusion criteria. Our initial literature search was conducted on Google Scholar using keywords such as 'knowledge of students on BC screenings'. We specifically targeted articles related to our primary group of interest — students in medical and related health sciences fields — with a focus on BC screenings. Key search terms included: 'medical/nursing/midwifery students', 'undergraduates', or 'future health professionals', 'BC screenings', 'prevention strategies', 'early detection', and 'breast-self-examination'. Subsequently, we conducted a search on PubMed, utilizing a query that included terms like 'medical students' or 'medical university undergraduates' and 'BC screening' or 'breast self-exam'. This search was refined with filters for female sex, English language, and publications after the year 2009. We removed duplicates identified through Google Scholar and added only those relevant to our criteria, corresponding to the second phase from Figure 1.

The following criteria were used to identify studies for this review: primary and secondary studies, published between January 2009 and August 2023, studies reporting evidence on female university students who study medicine or are related to medical fields such as nurses, midwives, pharmacists, public health and health sciences students, studies reporting evidence on BCS knowledge, attitude, practice, the perception among women, and articles in English. Exclusion criteria included studies focusing on health literacy, treatment, genetic counseling, awareness, studies reporting evidence on men, research published before 2009, studies published as reports, editorials, conference papers, book chapters, or reviews, research including other malignancies and articles generally focusing on cancer and having the target group college, high school, secondary school, and vocational school students, or students enrolled in other programs. We used the PRISMA-ScR template to report our review. Initially, we identified 980 records through Google Scholar and 229 through PubMed. However, we excluded certain articles for the following reasons: (a) inclusion of male students in the target group, n=15; (b) failure to specify the inclusion of only female participants in the target group, n=8; (c) focus on the effects of intervention/education sessions provided, n=6, (d) inclusion of non-medical students, n=10; (e) not including students in the target group, n=1, (f) unavailability of full text, n=2, (g) articles not available in English, n=6. Following these exclusions based on our eligibility criteria, the final analysis was conducted on 43 articles. This approach aligns with the third step of the PRISMA Flow Diagram, focusing on the selection process for the studies included in our review.

During the next step, researchers extracted and plotted the data. The data extraction framework included key elements such as the first author's name, publication date, study location and context, research design, objectives, study population, and main results (knowledge, attitudes, practices, perceptions, and awareness of BCS adoption). Two researchers independently evaluated each article, plotted information using the data extraction model, and adjusted the data extraction spreadsheet correspondingly.

## RESULTS

### BCS knowledge among female medical students

#### General knowledge

A study that aimed to evaluate medical students’ knowledge about BC symptoms and risk factors illustrated the belief that tobacco use, hormone replacement therapy, oral contraceptives, as well as not practicing breastfeeding are risk factors for BC [15]. In contrast, the risk factors most frequently identified by female students included in another study were family history (82.9%), not breastfeeding (68.0%), oral contraceptive use (58.6%), and late menopause (50.9%) [16]. However, less than half recognized early menstruation (45.9%) and a high-fat diet (40.5%) as risk factors. A study focusing on female medical students' understanding, attitudes, and behavior toward BSE found that all participants had a solid understanding of BC, with 95% believing that BSE may be a useful technique for early diagnosis. Approximately 85% of the participants believed early BC screening enhances survival rates. Additionally, 65% of students were familiar with all three BC screening techniques: MMG, CBE, and BSE. Specifically, 52.2% had heard of MMG, 48.90% believed it was done in a clinical laboratory, and even a smaller percentage (40.4%) knew it was recommended after age 35. In addition, 66.7% of the participants reported that early BC screening is important [17]. Similar points were highlighted in another study [18], where 15.5% of respondents had a strong general understanding of BSE. Additionally, a similar level of good general knowledge about BSE was observed among third and fourth-year nursing students in a separate study [7]. Moreover, female nursing students were well-informed about BC screening techniques and practiced BSE [12]. Also, 94.4% of participants in another study recognized that early detection of BC can improve outcomes [15]. Overall, good knowledge was present in other studies as well, where all participants were aware of BC, 80% considered it a common disease [19], and 65.3% were familiar with the BSE technique, although 19% did not know the technique and 15.7% were uncertain [20]. However, a gap between knowledge and practice was also seen, as 45.1% knew about BSE, whereas only 33.3% performed it [17]. Likewise, 91% of students believed that BSE is essential, but only 87% of students could perform it [21]. Similarly to the finding that most participants (71%) had BSE-related knowledge, 60% knew how BSE must be performed, but only 16% performed it regularly [15].

#### Level of education and knowledge

Higher educational levels in medical studies have been linked to increased knowledge about BC screenings. For instance, a study that assessed how education affects the understanding and use of BSE among female medical students in the preclinical phase compared to female medical students in the clinical period showed that those in the clinical phase demonstrated a better understanding and more accurate practice of BSE. This highlights how strong education may affect appropriate levels of knowledge and perhaps result in optimal levels of BSE practice [22]. Similarly, in another study, many first-year and second-year students had low knowledge levels (28.9% and 10.3%), whereas fourth-year and third-year students had high knowledge levels (8.2% and 4.1%). Nevertheless, a study conducted in Turkey targeting third and fourth-year nursing students found that BSE and CBE were reasonably low, and the difference between the two groups was statistically negligible even though both groups had extremely high BSE knowledge. However, there was a statistically significant difference between the study year and the confidence level. Fourth-year students demonstrated greater confidence in performing BSE than third-year students [7]. Also, there was a highly statistically significant relationship between age groups and level of education with the total mean knowledge of BSE because 60% of the study population in the study were younger nursing students (first and second year) who did not yet get an in-depth BC curriculum [18]. Moreover, the level of study was a significant predictor of BC screening knowledge. Third-year students were 14 times more likely to have a strong understanding of BC screening techniques compared to first-year students (OR=13.9, 95% CI, 6.2–31.26) [12, 23]. Another study found that clinical exposure significantly improved female medical students' comprehension of BC signs, risks, and confidence in identifying warning signs [24].

Differences in knowledge were also influenced by the field of study. For example, medical students were more likely to recognize that breastfeeding reduces BC risk compared to students in pharmacy (62.5%), dental sciences (86.7%), and nursing sciences (70.0%), who showed uncertainty about this association [25]. Another study identified that students enrolled in clinical nutrition, health administration, and laboratory medicine departments showed differences in their awareness of BC symptoms: 36% of the health administration students believed that changes in breast shape and size are the most common signs of BC [26]. Likewise, 28% and 37% of laboratory medicine and clinical nutrition students reported a lump or a moving mass as the most frequently encountered sign. Another study showed that having a college education is not a predicting factor for BSE knowledge, as more than half of the 20-year-old female participants included did not know about BSE [23]. This finding aligns with another study where participants showed poor knowledge of certain BC signs and symptoms [27]. However, no significant associations were found between participants' knowledge and factors like marital status, residential area, faculty department, and academic year [28].

#### Jobs and knowledge

Health professionals shared common views regarding the importance of BC screening. Specifically, 85 doctors (n = 1,005) were aware of BSE, reporting its usefulness for early detection of BC [29]. Comparing midwives with midwifery students, the rate of performing BSE was higher among midwives (52.5% practicing BSE monthly) compared to students (45.4%) [30]. There was no difference in the technique used when performing BSE. Most students used their contralateral arm, yet <17% had two or more different positions, such as standing and raising their hands or lying and pressing against the waist. Most participants, both midwives and students, had a positive attitude and opinion concerning the evolution and success rate of detecting BC and treatment in the future. Likewise, most were willing to receive additional information, education, and support in performing BSE [31]. However, despite knowledge of BSE practice, some students did not perform it regularly [32].

#### Knowledge regarding the time of practicing BSE

Medical students from Baghdad University in Iraq demonstrated a higher level of knowledge about the appropriate timing for performing BSE compared to their colleagues from Haramaya University, Ethiopia. For instance, 67 female medical students from Baghdad University (44.67%) in the pre-clinical category and 77 students (51.33%) in the clinical group correctly identified that BSE should be performed five days following menstruation. Also, 85 students (56.65%) in the preclinical group recognized that the BSE should be conducted every month, compared to 116 students (77.33%) in the clinical group [22]. Contrastingly, fewer medical students, 34 (26.9%) from Haramaya University, Ethiopia, thought it should be performed a few days after menstruation, which is the recommended time according to existing guidelines, compared to 51.5% of the participants who thought that BSE should be performed a few days before menstruation [8]. Nonetheless, most nursing students from an Indian university had good knowledge regarding the time of practice but not the starting age. For instance, according to 50.4% and 56.3% of respondents, BSE must be performed monthly using the palm and a minimum of three fingers, and 47.5% said that BSE should be performed even <19 years, although the recommended time is after 20 years [19]. Limited knowledge was also reported among nursing students from Riyadh, Kingdom of Saudi Arabia, as 78% were unaware of the proper timing for executing BSE or its frequency (72%) [33]. Unsatisfactory knowledge of the frequency and the ideal time to perform BSE among final-year medical students were identified among students from Sudan as well [34]. Additionally, their findings regarding BSE knowledge were unexpected, as the target group consisted of final-year female medical students, with the main assumption that students should have acquired this knowledge within the educational training.

#### Sources of knowledge

A significant proportion of medical students (46.7%) in one study learned about BSE for the first time through seminars, classes, and tutorials, which highlights the reality that strong education may favorably affect high levels of knowledge and possibly result in high levels of BSE practice [22]. Along the same lines, another study found that 54% of medical students heard of BSE through lectures as their primary source of information. In addition, radio and television combined (35.7%) were significant in raising awareness about BC [8]. Likewise, the majority (73.2%) of the study sample claimed to have learned about BSE at school [30, 35], followed by printed materials (39.4%), medical personnel (30.7%) [33, 36], TV/internet (34.6%), and acquaintances (3.9%) [7]. The sources of knowledge of nursing students from a Nigerian hospital also ranged from official coaching (45.1%), media (33.3%), and individual learning (21.6%) [12]. Contrary to previously published studies, it was found that mass media was the main source of BSE information [18]. Mass media (TV, radio, and newspapers) made up the majority of information sources (35.1), as suggested by other studies as well [19, 37, 38], followed by contacts with medical staff (9.3%), family (2.1%), and other sources (14.4%). The two most frequent sources of BC knowledge, according to students, were media (TV/radio) and seminars on health topics (46.3% and 42.2%, respectively) [39]. Similar to the study conducted among medical students from Kenya, media, including television and radio, were the main sources of BSE-related information, as mentioned by 65% of the participants, whereas only 7% mentioned health professionals [25]. Nevertheless, acquaintances can also be a source of knowledge among those aware of BSE, 35.2% learned from family, friends, or teachers, 20.4% from communication tools and media, 21.2% from healthcare professionals, 14.4% from the internet, and 8.3% from articles and books [20].

#### Knowledge regarding symptoms and signs

Participants agreed that a palpable breast lump should be the first sign of BC (77.7%), followed by nipple drainage (7.1%), breast discoloration (9.5%), and a shift in breast dimensions (3.17%). However, over 65% of people claimed that MMG, CBE, and BSE can detect BC [8]. A stratified random cross-sectional study found that out of 423 female medical students, 35.2%, 14.7%, and 35.9% had no information about the causes and risk factors, early manifestation, and early BC detection methods [40].

#### Attitudes of female medical students regarding BCS

We detected a favorable attitude towards BSE in a majority of studies. Particularly, 63.9% of respondents mentioned that all women should participate in BSE, but 4.2% claimed BSE makes people feel embarrassed, and 29.9% said they were afraid to think about BC [17, 41]. Generally, more than three-quarters (75.3%) of nursing students at an Arab American University had a positive attitude towards BSE, whereas about 21.6% were not interested in participating in BCS, with no reports of unfavourable attitudes [18]. The findings revealed that most students held a positive attitude towards BSE, and 93.3% thought that performing BSE is required, but fear of developing BC in the future was also the primary driver behind BSE (84.8%) [19, 40]. Positive attitudes were related to being knowledgeable about BSE, seeking medical assistance in case of an abnormal breast or lump notice, and the monthly practice of BSE [42].

### The practice of female medical students regarding BCS

#### Practice tendencies

The level of practice among students was generally low. For example, only 110 medical students (36.67%) from Baghdad University were practicing BSE, with 77 students (51.33%) in the clinical group never engaging in it [22]. Likewise, in another study, 62.9% of the students said they do not practice BSE monthly, and 42 students (43.3%) claimed that they had never been taught BSE by health professionals and had not studied the proper method for performing it [18]. This view was also supported by another paper, where the majority of participants (63.3%) refused to answer questions about the correct and complete method of performing BSE [22]. Another study [43] identified that 77.9% of female adolescents never engaged in any form of breast examination.

Despite the high percentage of public health students (89%, n=178) from Jimma University, Ethiopia, who had good BSE knowledge, only 21% (n=42) practiced it correctly. On the other hand, 25.5% (n=51) practiced it incorrectly, and more than half (107 students) did not practice it at all [44]. Furthermore, regardless of a good level of BSE knowledge, only 13.5% of monthly regular BSE cases and 10.3% of occasional CBE cases were detected among third and fourth-grade nursing students [7]. Only 33.3% routinely performed BSE throughout the year (12 times), and very few nursing students conducted an accurate and complete BSE [19]. Nevertheless, the existence of a physician in the family environment and the year of learning were both important mediators of BSE practice, according to multivariate analysis using binary logistic regression, which showed that about 90% of those who practiced BSE did so at least once a month [12].

#### Reasons for not performing BSE

Regarding the practice of BSE, 37.4% of medical students in Sudan said that they do not practice it because of negligence, whereas 26,2% do not know how to do it [45]. Lack of knowledge was also a barrier for female nursing students [19], and 20.8% of medical students relied on their poor performance due to the lack of familiarity with the technique [40]. There was a highly statistically significant correlation between BSE knowledge and practice, demonstrating the public’s willingness to get accurate BSE knowledge [18]. This aligns with findings from another study where students faced challenges accessing BSE information and lacked recommendations from others [46]. However, despite high levels of BSE knowledge, barriers to undergoing BSE were the lack of requirement to undertake the examination continuously, feeling healthy, and not having breast issues [37]. Other reasons included the lack of symptoms and the belief of not having BC [43], in line with about 45.7% of individuals who reported poor BSE performance attributed to the lack of breast issues [40]. Absence of symptoms or warning signs (28.8%), anxiety about seeing an anomaly (16.4%), and loss of intimacy (15.4%) were also causes for not performing BSE [8]. Also, a small percentage of the sample (4%) identified the absence of symptoms as a barrier to BSE practice [33]. This contrasts with the finding of another study [47], where 84.5% of the sample mentioned the importance and necessity of performing BSE despite the lack of symptoms. Another rationale cited as a cause for not practicing BSE was the failure to recall [7] (6.36% of individuals, as mentioned by two studies [35, 44]). Forgetfulness was mentioned by 17% of respondents [8] compared to 36.7% [36]. Not considering it necessary was also suggested by several studies. Specifically, 4.54% of medical students [21], contrasting to 13.3% of respondents, thought BSE was unnecessary and expressed fear of receiving a BC diagnosis [19]. This finding is similar to another study where 11.8% disagreed that BSE was essential [48] and did not consider it useful [49]. Lack of time was a commonly reported issue [49], mentioned by 22.0% of respondents in one study [36] and 11.5% in another [48]. A smaller proportion cited fear of the unknown [36], fear of detecting anomalies (2.3%), lack of expertise (22.7%), and lack of courage (6.8%) as reasons for not practicing BSE [48].

### Female medical students’ perceptions of BCS

Risk perception, an essential component of the behavioral change framework, significantly impacts women's decisions to undergo breast cancer testing. Women who either did not perceive themselves at risk for BC or were unaware of their risk status were less likely to consistently perform BSE compared to those who had a pessimistic view of their risk of developing the illness. Our findings support the Health Belief Model, which suggests that women with a greater likelihood of developing BC are more likely to do routine BSE, as they view the disease as a severe danger, have fewer perception obstacles, and a greater perception of the advantages [10]. Similarly, it is believed that frequent BSE testing should consider women's health views. Another study used the Health Belief Model Scale (HBMS) to determine students' perceptions of BC screenings. A score close to five indicates susceptibility, paying attention, and health motivation. Likewise, it indicates that the advantages, barriers, and self-efficacy of BSE are highly acknowledged. The students' HBMS importance, health interest, and BSE value perception ratings were not significantly different from their BC knowledge and BSE competence scores across different academic years. Compared to students who participated in BSE infrequently or not at all, those who participated in BSE regularly perceived their vulnerability as being lower. Their BSE self-efficacy was stronger, their perception of BSE barriers was smaller, and a statistical difference was discovered [7]. Also, most studies indicated that medical students' perceptions of the major BC risk factors were generally positive [8].

## DISCUSSION

The objective of this scoping review was to explore the existing research on the knowledge, attitudes, practices, and perceptions of female medical students and those in related medical fields regarding BCS, to influence public health and policy efforts, and to enhance screening procedures. It is important to analyze this paper in terms of the strengths and limitations it encompasses. Our research provided information on the level of knowledge, attitudes, and practices related to BCS among female medical students. The study offered broad perspectives, including a comprehensive analysis of 43 published scientific articles. On the other hand, as we limited our analysis to articles written only in English, this might impact the generalizability of our results, which could be an apparent limitation of our study.

We suggest utilizing the media and other channels to increase public awareness of the significance of BSE. First, appropriate health education initiatives are required to explain the risk factors and prevention of BC to medical students and the general population [8]. Intensified awareness projects and implementation of health campaigns among nursing university students are also required to supervise their current level of BC and BSE knowledge. Effective and coordinated implementation of awareness and health education programs can lead to beneficial behavioral changes. The use of media as a key tool for disseminating BSE information is essential for increasing public awareness [18]. These results agree with a study underlining the importance of promoting BSE awareness [19]. The study suggests that consistent information delivery and maintaining BSE practice can be improved with reminders and notifications. A unique and beneficial approach for students could be using a smartphone in conjunction with practical experience for BSE education. We suggest this teaching strategy be more widely applied to young women [46]. Therefore, the public must be actively informed about BSE and BC via the media. Medical professionals must use media more accurately and effectively [20].

Improving the current curricula for medical, midwifery, and nursing students should also be applied. For example, one study suggested that, as a substitute for regular training, specific training sessions need to be organized to enhance students' understanding, abilities, practices, and health convictions regarding BSE. The curricula should be updated to include information about BC [7]. In undergraduate nursing school curricula, changes are needed to support nurses' increasing involvement in cancer prevention screening. These reforms should encompass nurse education and training [35, 30, 46, 47]. Several studies have shown significant improvements in knowledge following educational activities. Specifically, there was a notable increase in the understanding of BCS techniques (i.e., CBE and BSE) among both basic science nursing (Group 1) and general nursing and midwifery students (Group 2) after educational interventions, compared to their initial knowledge levels. Moreover, following the presentation, 89% (n=31/35) of students in Group 2 and 96% (n=54/56) in Group 1 were prepared to catalyze the society for BC testing [11]. In addition, there was a statistical difference between the pre- and post-education programs for 67.3% of students [50]. However, in terms of attitudes towards BC and knowledge and proficiency in performing BSE, there was no statistically significant change in scores observed between the pre-education, 6-month, and 1-year follow-ups of the students [51, 52].

## CONCLUSION

This paper identified that the majority of students had good general knowledge regarding signs, symptoms, and early detection measures. Their information sources included family, friends, media, and TV, mainly classes and lectures. Most of them had a positive attitude and expressed an optimistic belief regarding BC preventive measures. Despite these, their practice level was poor. This supports the necessity of addressing this topic and finding a proper solution. Although the study successfully explored the existing literature on female medical students' knowledge, including signs and symptoms of BC and techniques of BSE, which are important to improve the detection of BC, it had certain limitations that need to be acknowledged. One limitation of the study is that it relied only on two databases, PubMed and Google Scholar, to identify scientific articles. Nevertheless, our analysis presented valuable insights into BCS, but more research is required to determine the optimal course of action for enhancing the adoption and application of BSE and other early BC detection techniques.
